# Indoor falls and number of previous falls are independent risk factors for long-term mortality after a hip fracture

**DOI:** 10.1007/s40520-023-02551-3

**Published:** 2023-09-09

**Authors:** Montserrat Barceló, Jordi Casademont, Jordi Mascaró, Ignasi Gich, Olga Herminia Torres

**Affiliations:** 1 Geriatric Unit, Departament de Medicina, Internal Medicine Department, Hospital de La Santa Creu I Sant Pau, Universitat Autònoma de Barcelona, Mas Casanovas Street, no. 90, 08041 Barcelona, Spain; 2 Department of Clinical Epidemiology and Public Health, CIBER Epidemiología Y Salud Pública (CIBERESP), HSCSP Sant Pau Biomedical Research Institute (IIB Sant Pau), Barcelona, Spain

**Keywords:** Mortality, Hip fracture, Falls

## Abstract

**Background:**

Hip fractures are almost always the result of a fall. Causes and circumstances of falls may differ between frail and vigorous patients.

**Aim:**

To describe the circumstances of falls causing hip fractures, number of falls during the previous year, and their association with long-term mortality.

**Patients and methods:**

The study is a retrospective review conducted in a tertiary university hospital serving a population of 425,000 inhabitants in Barcelona. All patients admitted with hip fractures with medical records describing the circumstances and number of previous falls were included. The number of falls in the previous 12 months was recorded, including the one causing the fracture. The circumstances of the index fall were dichotomized according to whether it was from the patient’s own height or above; day or night; indoors or outdoors, due to intrinsic or extrinsic causes. Cumulative mortality was recorded for almost 5 years after hip fracture.

**Results:**

Indoor falls were strongly associated with shorter survival. Falling more than once in the previous year was also a risk factor for long-term mortality (hazard ratio 1.461, *p* < 0.001 and hazard ratio 1.035, *p* = 0.008 respectively).

**Conclusion:**

Indoor falls and falling more than once in the previous year are long-term risk factors for mortality after hip fractures. It is always essential to take a careful patient history on admission to determine the number of falls and their circumstances, and special care should be taken to reduce mortality in patients at high risk.

## Introduction

It is well known that the short- and long-term mortality following a hip fracture is high [1]. The risk factors for mortality have been extensively studied in the literature. Some, such as advanced age, male sex, and comorbidity, have been described in virtually all studies [2, 3]. Frailty has also been suggested as a possible risk factor for mortality [4] although this is difficult to measure once the hip fracture has occurred.

Hip fractures are almost always the result of a fall [5]. A study by Norton et al. found that 96% of hip fractures were associated with a fall [6]. Falls are also more common in frail older adults. In another study, Speechley et al. observed that the incidence of falling in frail patients in one year of follow-up was 52%, but they also found that falls in vigorous older adults resulted in more serious injuries [7]. Some studies have shown that causes and circumstances of falls differ between frail and vigorous patients. Vigorous patients were more likely to fall during ambulatory activities, when there was an environmental hazard, and on stairs [7, 8]. This difference in fall patterns has also been observed in falls that result in hip fracture [9, 10]. Given that frail patients have an increased risk of mortality when an adverse event such as a hip fracture occurs [11], there may be some relationship between how the fall that caused the hip fracture occurred and the risk of mortality.

The aims of our study were to analyze whether the characteristics of a fall that caused a fracture and the number of falls in the previous year were related to long-term mortality in the first 5 years after a hip fracture.

## Patients and methods

### Setting

The study was carried out in the Department of Orthopaedic Surgery of a tertiary university hospital serving a population of about 425,000 inhabitants in Barcelona, Spain.

### Subjects

The clinical data of all patients admitted to the Orthopaedic Surgery Unit with a diagnosis of hip fracture between December 2009 and September 2015 were retrospectively reviewed. Inclusion criteria: the circumstances of the fall that led to hospital admission and the number of falls in the previous year had been recorded in the medical records, and patients for whom the date of death was known or whether they were still alive at least 5 years after hospital discharge. Patients who died during hospital admission were included. Patients with hip fractures due to accidents or neoplasm (bone metastases) were excluded.

### Data collection

The main risk factors for mortality collected were: age, sex, comorbidity, measured by the Charlson index [12], the Barthel Index of Activities of Daily Living (ADL) [13], number of drugs before admission, place of residence, length of hospital stay, hemoglobin and albumin at admission, inpatient rehabilitation center at discharge, and traumatological and medical complications, including blood transfusion and delirium.

The number of falls in the previous 12 months, including the one that caused the fracture, was recorded. The circumstances of the index fall were dichotomized according to whether it was from the patient’s height or above, during the day or night, indoors or outdoors, and whether it was due to intrinsic or extrinsic causes. In our environment, some authors define daytime as between 8 a.m. and 8 p.m. [14]. We considered daytime as between sunrise and sunset, as daylight hours in our country vary considerably between winter and summer. Intrinsic causes were those due to age-related changes, neurological diseases (gait impairment, Parkinson’s disease, cognitive impairment, polyneuropathy, and stroke sequelae), diabetes, postural hypotension, osteoarthritis, vascular diseases, foot problems, sarcopenia or myopathies, visual or auditory impairment, or symptoms, such as dizziness, weakness or vertigo, and acute illnesses. Extrinsic causes were those considered to be environmental hazards (poor lighting, slippery floors, footwear, home or neighborhood features), or drugs and alcohol use. Some authors regard medication as an intrinsic factor [15]; here, following Phelan et al. [16], it was considered an extrinsic factor, as medication is usually a modifiable environmental factor, and most intrinsic factors are not, or are only partially modifiable.

### Hip fracture pathway

Our hospital has an established hip fracture pathway. Consequently, during admission, all patients are visited at least once by a physician from the Geriatrics Unit for a geriatric assessment, in accordance with the co-operation framework on hip fractures developed between the two departments. All patients (or their relatives) were asked by the attending physician about the number of falls in the previous year, and the characteristics of the fall that led to admission. In some patients, it was not possible to collect complete data due to cognitive impairment and lack of witnesses.

### Mortality after discharge

Retrospectively, we collected the date of death of the patient or whether they survived for at least 5 years after hospital discharge, although in some patients it was possible to obtain data for up to 10 years after admission. The date of death was obtained from the patient’s medical records and the National Death Index website of the Ministry of Health (https://www.mscbs.gob.es/estadEstudios/estadisticas/estadisticas/estMinisterio/IND_TipoDifusion.htm), subject to prior authorization.

### Statistical analysis

Continuous variables were described by calculating means and standard deviations, then compared using the Student’s *t*-test with the Welch correction. For categorical variables, absolute numbers, relative frequencies, or proportions were calculated and compared using Fisher’s exact test. Multivariate Cox regression analysis (proportional hazards model) was performed to determine independent variables for mortality five years after sustaining a hip fracture. A Kaplan–Meier survival analysis over the follow-up period was calculated. Statistical analysis was performed using the IBM SPSS Statistical Package (version 26) (SPSS Inc., Chicago, IL, USA) [17]. Statistical significance was *p* ≤ 0.05 in all cases.

## Results

Between December 2009 and September 2015, 2909 patients were admitted to hospital with hip fracture. Complete data of the circumstances of the index fall and number of previous falls were obtained for 1364 (47.1%) patients, and 1349 (98.9%) of these were located five years after hip fracture. Cumulative mortality was 266 (19.7%) at one year, and 755 (56%) at five years.

**Fig. 1:**
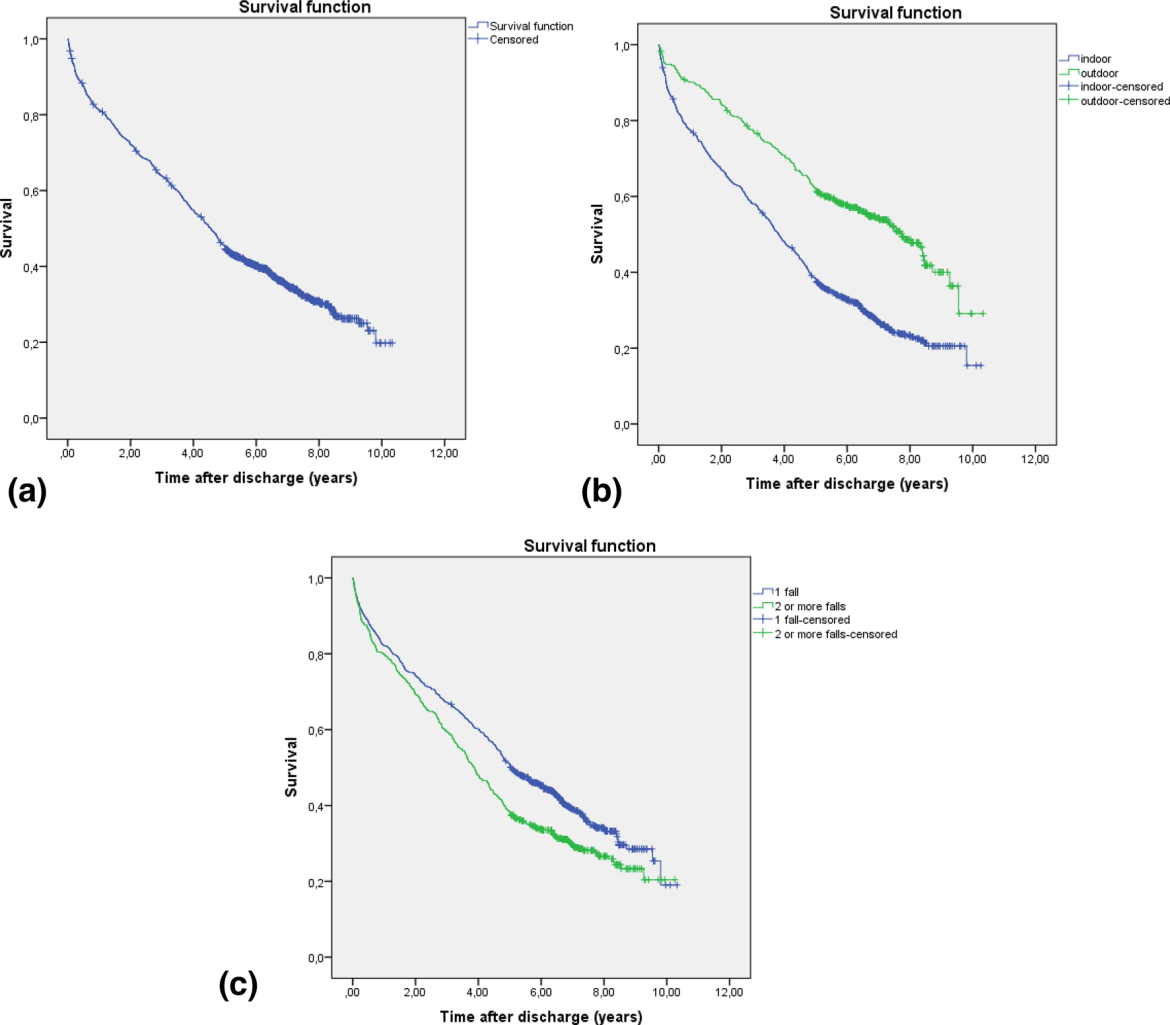
Kaplan–Meier curves for cumulative survival after hip fracture. **a** Cumulative survival in relation to all parameters. **b** Cumulative survival in relation to an indoor/outdoor index fall. **c** Cumulative survival in relation to more than one fall in the previous 12 months, including the index fall

Baseline characteristics at admission and in-hospital outcomes are shown in Table 1, highlighting a high mean age, predominance of female sex, and a high number of medical complications, the most frequent being respiratory infections (11.3%), heart failure (4.9%), urinary infections (13.6%), and acute renal failure (13.6%). Many patients have more than one complication during admission. The mean number of falls was more than two, most occurred indoors and were due to intrinsic factors, and only a very small number occurred during the night or from a height. Characteristics of surviving and non-surviving patients at 5 years are compared in Table 2, all were statistically significant except for traumatological complications and discharge to an inpatient rehabilitation center. Non-survivors had more falls in the previous year, and fell more indoors, due to intrinsic factors, at night and from their own height. Multivariate Cox regression analysis for independent variables associated with mortality one year and five years after sustaining a hip fracture is shown in Tables 3 and 4. The risk factors with the strongest association with short- and long-term mortality were advanced age, male sex, higher comorbidity, delirium and other medical complications during admission, and falls indoors. A higher number of falls were a risk factor only in the long term. A higher Barthel index, and hemoglobin and albumin at admission were also statistically significant protective factors, although the association was small. Living at home was a protective factor, but only in the long-term. The Kaplan–Meier curve shows the estimated probability of survival after hip fracture during a mean follow-up of 4.24 years, as some patients died before 5 years shown in Fig. 1a, b, which compares the post-discharge survival over time between patients who fell indoors versus those who fell outdoors, while Fig. 1c compares long-term survival after discharge between patients who sustained one fall versus those who sustained two or more falls in the previous year.

**Table 1:** Baseline characteristics, in-hospital results, and circumstances of the fall

	*n* = 1349
Mean age in years (SD)	83.83 (8.78)
Female sex* n* (%)	1050 (77.8)
Barthel index (SD)	82 (19.75)
Charlson index (SD)	1.27 (1.42)
Dementia *n* (%)	174 (12.9)
Number of drugs (SD)	6.18 (3.47)
Place of residence, *n* at home (%)	1159 (85.9)
Length of hospital stay, in days (SD)	12.26 (7.23)
Hemoglobin at admission, in g/L (SD)	122.27 (16.54)
Albumin at admission, in g/L (SD)	29.36 (3.64)
Delirium* n* (%)	203 (15)
Blood transfusion *n* (%)	657 (48.7)
Other medical complications *n* (%)	477 (35.4)
Traumatological complications *n* (%)	30 (2.2)
Number of falls (previous 12 months)* (SD)	2.15 (2.39)
Indoor fall *n* (%)	950 (70.4)
Intrinsic factors *n* (%)	1031 (76.4)
Daytime fall *n* (%)	1237 (91.7)
Own height* n* (%)	1309 (97)
Discharge to inpatient rehabilitation center *n* (%)	790 (58.6)

*SD* standard deviation

*Including index fall

**Table 2:** Comparison of survivors and non-survivors at 5 years

Patients = 1349	Exitus *n* = 755	Survivors *n* = 594	*p*
Mean age in years (SD)	86.40 (7.3)	80.57 (9.41)	< 0.001
Female sex *n* (%)	544 (72.1)	506 (85.2)	< 0.001
Barthel index (SD)	76.80 (20.7)	88.57 (16.25)	< 0.001
Charlson index (SD)	1.58 (1.56)	0.88 (1.1)	< 0.001
Dementia *n* (%)	124 (16.4)	50 (8.4)	< 0.001
Number of drugs (SD)	6.52 (3.58)	5.75 (3.28)	< 0.001
Place of residence, *n* at home (%)	618 (81.9)	541 (91.1)	< 0.001
Length of hospital stay in days (SD)	13.03 (7.54)	11.28 (6.7)	< 0.001
Hemoglobin at admission in g/L (SD)	119.83 (17.5)	125.38 (14.67)	< 0.001
Albumin at admission in g/L (SD)	28.75 (3.62)	30.14 (3.53)	< 0.001
Delirium *n* (%)	158 (20.9)	45 (7.5)	< 0.001
Blood transfusion *n* (%)	413 (54.7)	244 (41.1)	< 0.001
Other medical complications *n* (%)	336 (44.5)	141 (23.7)	< 0.001
Traumatological complications *n* (%)	21 (2.8)	9 (1.5)	0.082
Number of falls (previous 12 months)* (SD)	2.39 (2.76)	1.86 (1.78)	< 0.001
Indoor fall *n* (%)	599 (79.3)	351 (59.1)	< 0.001
Intrinsic factors *n* (%)	618 (81.9)	413 (69.5)	< 0.001
Daytime fall *n* (%)	683 (90.5)	544 (93.3)	0.039
Own height *n* (%)	744 (98.5)	565 (95.1)	< 0.001
Discharge to inpatient rehabilitation center *n* (%)	450 (59.6)	340 (57.2)	0.206

*SD* standard deviation

*Including index fall

**Table 3:** Risk factors for mortality at 1 year based on multivariate Cox regression analysis

	Hazard ratio	95% confidence interval	*p*
Age	1.038	1.019–1.058	< 0.001
Male sex	2.094	1.595–2.749	< 0.001
Charlson index	1.131	1.045–1.225	0.002
Barthel index	0.992	0.986–0.997	0.004
Hemoglobin at admission	0.990	0.983–0.997	0.006
Albumin at admission	0.941	0.908–0.975	0.001
Delirium	1.546	1.154–2.071	0.004
Other medical complications	1.417	1.097–1.831	0.008
Indoor falls*	1.784	1.256–2.534	0.001

*Including index fall

**Table 4:** Risk factors for mortality at 5 years based on multivariate Cox regression analysis

	Hazard ratio	95% confidence interval	p
Age	1.055	1.044–1.067	< 0.001
Male sex	2.051	1.746–2.410	< 0.001
Charlson index	1.165	1.111–1.223	< 0.001
Barthel index	0.992	0.989–0.996	< 0.001
Place of residence at home	0.794	0.661–0.954	0.014
Hemoglobin at admission	0.991	0.987–0.995	< 0.001
Albumin at admission	0.958	0.938–0.978	< 0.001
Delirium	1.386	1.161–1.656	< 0.001
Other medical complications	1.246	1.080–1.437	0.003
Indoor falls*	1.461	1.233–1.732	< 0.001
Number of falls (previous 12 months)*	1.035	1.009–1.062	0.008

*Including index fall

## Discussion

In our study, the risk factors for long-term mortality after a hip fracture were a fall sustained indoors and more than one fall in the previous year, including the index fall, together with other risk factors for mortality previously described in the literature. These data can be easily obtained by asking simple questions that should be asked of all patients admitted to hospital following a fall; indeed, the recent World Guidelines for falls prevention and management for older adults has emphasized, among other things, the importance of asking for details of the event and previous falls [18].

In our patients, we found an association between mortality and a fall sustained indoors, from the height of the patient, at night, and due to intrinsic factors; after multivariate analysis, only the place of the index fall (indoors), remained an independent risk factor of mortality. Falling indoors is strongly correlated with lower survival, since the trend continues throughout the entire period, and even ten years after discharge. To our knowledge, the only previously reported study that directly relates the causes and circumstances of a fall to mortality after hip fracture is a paper by Burm et al. with an average follow-up of 1.5 years; the same authors also found that indoor falls had higher mortality than outdoor falls [19]. Other studies link indoor falls to poor physical function although they do not directly correlate with mortality after hip fracture [10, 11]. Some papers have pointed out that even if an indoor fall does not cause a fracture, it may signal functional decline in older adults [20] and indoor fallers show a significant excess of mortality [21]. Although indoors are where most falls occur [5], it is also true that frail older patients spend more hours at home than more active ones [22].

Our study also showed that more than one fall in the previous year (including the index fall) was a risk factor for long-term mortality, but not for short-term mortality, and was associated with increased mortality over more than 5 years. Unsurprisingly, mortality was higher in recurrent fallers, as falls have frequently been linked to greater frailty and increased mortality [7, 23, 24].

We have not found any studies that associated other circumstances of the index fall (falls from the patient’s height or above, during the day or night, and due to intrinsic or extrinsic causes) to mortality. We were surprised that falls due to intrinsic factors were not risk factors for mortality, as those due to extrinsic factors have been related to better levels of physical function than those with physiological causes [9]. In our study, the number of patients who fell due to intrinsic factors was very high (more than three-quarters of the index falls) and very few patients fell from a height, such as a ladder. This may be one of the reasons why this circumstance was not significant in multivariate analysis.

Frailty has been associated with increased hospitalizations, higher rates of falls, hip fractures, admission to institutions and all-cause mortality [25–27]. Assessment of frailty in a patient admitted for a hip fracture is always complicated as we cannot use physical measures such as gait speed. Some authors have used wrist strength, as muscle weakness is considered a key element in frailty [28, 29], but there is still no standardized method. Other methods of studying frailty such as the FRAIL scale [30] have the disadvantage that, when it comes to subjective questions such as fatigue, proxy scores are no replacement for self-reported frailty [31]. Speechley et al. found that vigorous patients fell more often during activities involving walking about, when there were environmental hazards present, and on stairs, and that their falls resulted in more serious injuries [7], while other articles point out that fall patterns in frail and non-frail patients are different [9, 27]. This suggests that obtaining a better picture of the circumstances of the fall could provide an indirect measure of the person’s frailty.

The rest of the risk factors for mortality found in our study have been described in previous studies and are very similar worldwide [1, 32]. The factors most associated with mortality are gender and advanced age. In studies carried out in Western countries, mortality in males can be up to twice as high as in women [3]. It is not known why mortality is higher in men, since women are at higher risk of falls, osteoporosis and frailty. Some authors have linked it to the higher frequency of infections in men. A study conducted by Wehren et al. found that mortality in men two years after hip fracture was twice as likely due a higher incidence of pneumonia, influenza, and septicemia [33]. Although in most studies, the mean age of men with hip fractures is lower than that of women, the comorbidity burden among men is higher and their health status is poorer, and men are more likely to die due to previous chronic diseases [34]. Older age is associated with higher mortality regardless of where the study was conducted, although the cut-off point at which there is an increased risk of death is unknown [34]. A study carried out in Spain found higher in-hospital mortality in hip fracture patients older than 90 years [35], and a paper by Australian authors described similar results [36]. A study in hip fracture patients older than 75 years found an overall mortality of 40% at one year, which was significantly higher in those older than 85 years. The authors concluded that age was the most important determinant of long-term mortality [37].

Other well-known risk factors confirmed in our study were higher comorbidity (with an increased risk for more than one comorbidity) [3], medical complications during admission, including delirium [38], or living in a nursing home [4], as has also been reported previously.

A higher Barthel index, and hemoglobin and albumin at admission were all protective factors in our study. Low hemoglobin at admission has been associated with higher in-hospital mortality [35], at 30 days, at one year [2, 39], and also in the long-term [40], this author indicates that only hemoglobin at admission, and not other controls during admission, is an independent predictor, and links anemia to advanced age, type of fracture and previous comorbidities.

Some studies have described albumin as a risk factor for in-hospital [41] and one-year mortality [42]. A study carried out in patients with intra-capsular fractures, found that albumin was an independent risk factor for long-term mortality (up to 9 years), and related it to previous poor health, in-hospital complications, as well as a marker of malnutrition [43]. However, in many studies, neither hemoglobin nor albumin has been described as a risk factor for mortality. This remains a controversial issue [3, 44].

Postoperative outcome prediction still needs the performance of traditional predictive statistical models because current implementations of artificial intelligence do not seem to provide substantial benefit for hip fracture mortality prediction [45].

Based on this study and others carried out at our hospital, efforts have been made to detect fragile patients, to implement measures to prevent complications during admission, such as geriatric and trauma teams with shared responsibilities, diagnosis of oropharyngeal dysphagia and follow-up by speech therapist, an intravenous iron therapy protocol to treat anemia to reduce transfusions, among others. These patients also receive advice aimed at preventing further falls.

The main limitation of our study is that it is a retrospective, single center study. Furthermore, it was not always easy to clarify the circumstances of the fall, although it should also be pointed out that the data were collected prospectively, according to protocol, by the geriatric physician at the first patient visit, and patients for whom the circumstances of the fall were not correctly recorded were excluded. Another limitation linked to the retrospective nature of the study is that those patients for whom it was not possible to obtain relevant information about the fall could also be the frailest, with cognitive impairment and living in nursing homes. The main strength of our study is that it includes a large number of patients with a long follow-up, and it was possible to locate the vast majority, at least during the first five years after discharge. To our knowledge, there are very few studies linking the number and circumstances of falls to mortality after hip fracture, and this is the first study in our environment.

## Conclusions

Falling indoors and the number of falls in the previous year are related to long-term mortality after hip fracture. Answers to simple questions, such as where the patient fell and the number of falls in the previous year, are useful, not only to help prevent future falls, but also to provide an indirect estimate of frailty and to better understand the patient’s mortality risk.

## Declarations

### Study approval

This study protocol was reviewed and approved by Ethics Committee of the Hospital de la Santa Creu i Sant Pau, approval number IIBSP-FEM-2020–111.
